# *QuickStats*: Percentage of Adults Aged ≥60 Years Who Ever Had the Shingles Vaccine,* by Sex — National Health Interview Survey, 2008–2016^^†^^

**DOI:** 10.15585/mmwr.mm6719a9

**Published:** 2018-05-18

**Figure:**
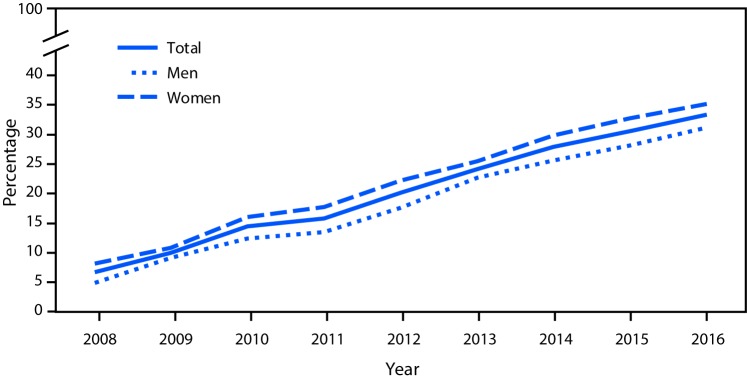
The percentage of adults aged ≥60 years who ever had the shingles vaccine increased from 6.7% in 2008 to 33.4% in 2016. The percentage of men who had the vaccine increased from 4.9% to 31.2%, and the percentage of women who had the vaccine increased from 8.2% to 35.2%. For each year during 2008–2016, women were more likely than men to have had the shingles vaccine.

**Source:** National Center for Health Statistics. National Health Interview Survey, 2008–2016. https://www.cdc.gov/nchs/nhis.htm.

